# Osteoclasts are not a source of SLIT3

**DOI:** 10.1038/s41413-020-0086-3

**Published:** 2020-02-19

**Authors:** Na Li, Kazuki Inoue, Jun Sun, Yingzhen Niu, Sarfaraz Lalani, Alisha Yallowitz, Xu Yang, Chao Zhang, Rong Shen, Baohong Zhao, Ren Xu, Matthew B. Greenblatt

**Affiliations:** 1 State Key Laboratory of Cellular Stress Biology, School of Medicine, Xiamen University, Xiamen, Fujian China; 2 Arthritis and Tissue Degeneration Program and David Z. Rosensweig Genomics Research Center, Hospital for Special Surgery, New York, NY USA; 3 Department of Medicine, Weill Cornell Medical College, Cornell University, New York, NY USA; 4 Department of Pathology and Laboratory Medicine, Weill Cornell Medical College, Cornell University, New York, NY USA; 5 Division of Adult Reconstruction and Joint Replacement, Department of Orthopedic Surgery, Hospital for Special Surgery, New York, NY USA; 6 Institute for Computational Biomedicine, Cornell University, New York, NY USA

**Keywords:** Bone, Metabolism

## Abstract

The axon guidance cue SLIT3 was identified as an osteoanabolic agent in two recent reports. However, these reports conflict in their nomination of osteoblasts versus osteoclasts as the key producers of skeletal SLIT3 and additionally offer conflicting data on the effects of SLIT3 on osteoclastogenesis. Here, aiming to address this discrepancy, we found no observable SLIT3 expression during human or mouse osteoclastogenesis and the only modest SLIT3-mediated effects on osteoclast differentiation. Conditional deletion of SLIT3 in cathepsin K (CTSK)-positive cells, including osteoclasts, had no effect on the number of osteoclast progenitors, in vitro osteoclast differentiation, overall bone mass, or bone resorption/formation parameters. Similar results were observed with the deletion of SLIT3 in LysM-positive cells, including osteoclast lineage cells. Consistent with this finding, bone marrow chimeras made from *Slit3*^*−/−*^ donors that lacked SLIT3 expression at all stages of osteoclast development displayed normal bone mass relative to controls. Taken in context, multiple lines of evidence were unable to identify the physiologic function of osteoclast-derived SLIT3, indicating that osteoblasts are the major source of skeletal SLIT3.

## Introduction

Drugs for the treatment of skeletal disorders, such as osteoporosis, typically fall into one of two categories. These drugs function to either block bone resorption by osteoclasts or augment bone formation by osteoblasts. However, current treatment options still have strong limitations, due in part to both the few anabolic agents available and restrictions on the use of these agents.^1,2^ To continue to advance therapy for disorders of low bone mass, the identification of new strategies to promote bone formation is critical.

Axon guidance cues have emerged as agents of great interest in this regard, as they control bone formation by a variety of mechanisms, including shaping the skeletal microenvironment to support osteogenesis.^3–7^ Among them, SLIT3 was recently identified as an osteoanabolic agent that ameliorated the bone loss in mouse models in two recent reports.^8,9^

SLIT3 was first discovered as a repulsive axon guidance cue during neuronal migration.^10^ SLIT3 is now increasingly recognized to play additional physiological roles outside of the nervous system, as it is involved in immunoregulation, stem cell regulation, and cancer development.^11–13^ More recently, SLIT3 was characterized as having angiogenic functions in nonbone tissues, and SLIT3 knockout mice displayed severe developmental vascular defects in the diaphragm.^14–16^ In vitro, recombinant SLIT3 stimulation could enhance angiogenesis by increasing the proliferation, migration and tube formation of endothelial cells.^15^ In contrast, SLIT3 signaling inhibition significantly decreased functional blood vessel formation in human engineered tissue.^17^

Our prior study showed that SLIT3 is highly expressed in osteoblasts, where it acts as a proangiogenic factor in bone to increase the levels of skeletal vascular endothelium and thereby increase bone formation. Through these angiogenic effects, recombinant SLIT3 was found to have therapeutic activity in mouse models of fracture healing and postmenopausal osteoporosis.^8,15^ Meanwhile, a separate study reported that SLIT3 is critical for skeletal physiology and also found osteopenia and a reduction in the skeletal vascular endothelium in *Slit3*^−/−^ mice.^9^ However, in this report, osteoclasts, as opposed to osteoblasts, were nominated as a key source of SLIT3 to control coupling between osteoblasts and osteoclasts. Given these conflicting data and the fundamental importance of SLIT3 as a promising osteoanabolic agent and physiologic signal linking bone metabolism to skeletal angiogenesis, further studies to clarify the cellular sources and targets of SLIT3 are needed.

## Results

### The expression of SLIT3 in osteoclastogenesis

**Fig. 1:**
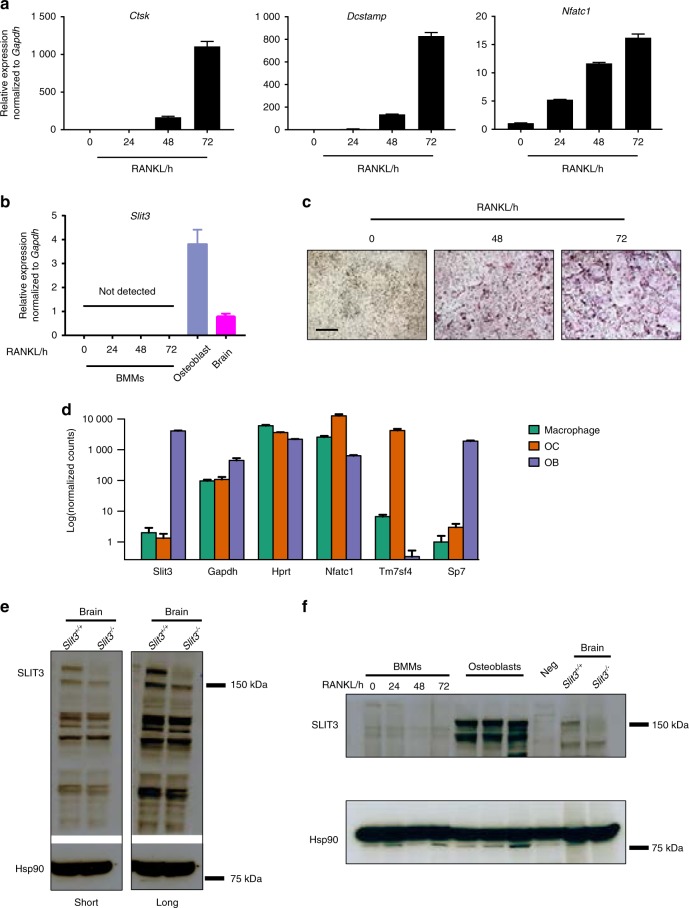
The expression of SLIT3 during osteoclastogenesis. **a**, **b** Quantitative real-time PCR analysis of mRNA expression of *Ctsk*, *Dcstamp*, *Nfatc1*, and *Slit3* in WT BMMs treated with RANKL for 72 h using osteoblast and brain tissue as positive controls (*n* = 3). **c** Representative TRAP-staining images of WT BMMs treated with RANKL for 0, 48, and 72 h (*n* = 4 total images per group). **d** Gene expression of *Slit3*, *Gapdh*, *Hprt*, *Nfatc1*, *Tm7sf4* (*Dcstamp*), and *Sp7* in RNA-seq analysis of primary macrophages, osteoclasts and osteoblasts derived from WT mice (*n* = 3). **e** Immunoblot analysis of SLIT3 expression in whole brain lysates from *Slit3*^*+/+*^ and *Slit3*^−*/*−^ mice. **f** Immunoblot analysis of SLIT3 expression in BMMs treated with RANKL for 48 h, with whole-brain lysates from *Slit3*^*+/+*^ mice and osteoblast lysates used as positive controls and whole brain lysates from *Slit3*^−*/*−^ mice and murine bone marrow-derived endothelial cells (Neg) used as negative controls

To clarify the cellular sources of SLIT3, we first examined *Slit3* expression in parallel with other osteoclast makers using real-time PCR during in vitro osteoclastogenesis. Osteoclast formation was further monitored by tartrate-resistant acid phosphatase (TRAP) staining (Fig. 1a–c). Unlike the robust *Slit3* expression observed in the brain and primary osteoblasts, we were unable to detect *Slit3* mRNA expression during bone marrow macrophage (BMM)-derived osteoclastogenesis at the mRNA level (Fig. 1b). To further confirm this observation, we analyzed RNA-sequencing (RNA-seq) transcriptional profiling data from macrophages, osteoclasts, and osteoblasts derived from wild-type mice. This approach also showed that *Slit3* expression in osteoclasts was negligible relative to its expression in osteoblasts. Western blotting using a polyclonal SLIT3 antibody validated for specificity (Fig. 1e) also failed to detect SLIT3 expression during osteoclastogenesis (Fig. 1f). These observations are consistent with those of prior analyses of axon guidance cue expression during osteoclast and osteoblast differentiation published by other groups.^5,18^ Moreover, SLIT3 expression was also not detected in prior studies of human osteoclast differentiation.^8,19^ Thus, we were unable to find evidence that SLIT3 is expressed in osteoclast lineage cells, which contrasts with the robust expression of SLIT3 observed in osteoblasts.^8^

### The effect of recombinant SLIT3 on osteoclastogenesis in vitro

**Fig. 2:**
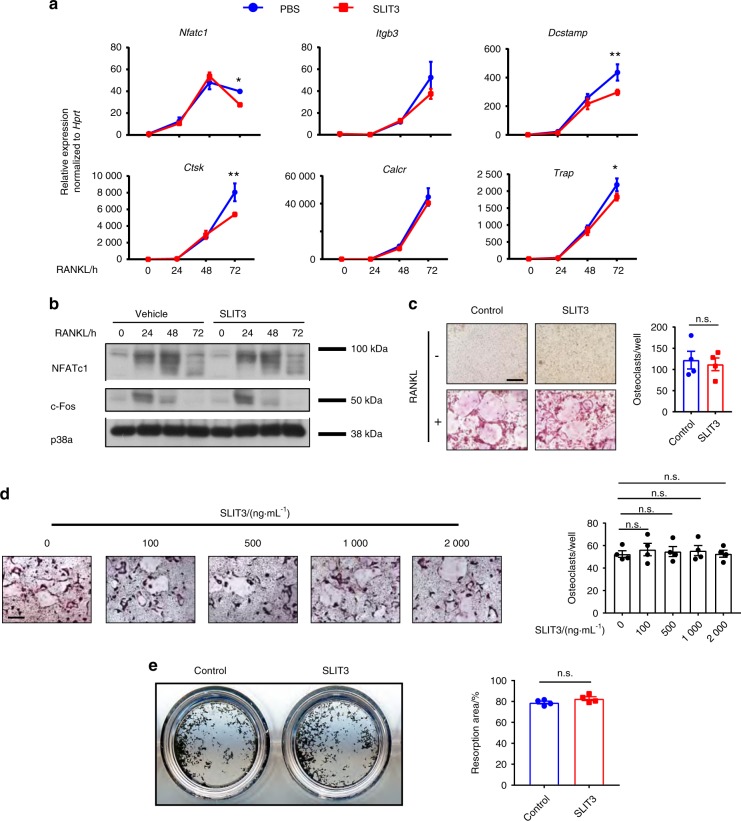
The effect of recombinant SLIT3 on osteoclastogenesis. **a** Quantitative real-time PCR analysis of the mRNA expression of *Nftac1, Itgb3, Dcstamp, Ctsk, Calcr*, and *Trap* in BMMs treated with RANKL and recombinant SLIT3 (1 μg·mL^−1^) for 72 h (*n* = 3). **b** Immunoblot analysis of Nfact1 and c-fos expression in BMMs treated with RANK and recombinant SLIT3 (1 μg·mL^−1^) for 72 h using p38a as a loading control. **c**, **d** Osteoclast differentiation using WT BMMs stimulated with RANKL and recombinant SLIT3 for 72 h. TRAP staining (left panel) was performed, and the area of TRAP-positive cells (nuclei/cell ≥3) per well relative to the WT control was calculated (right panel) (*n* = 4). **e** Mineral resorption activity of WT BMMs cultured on calcium-coated plates with RANKL and recombinant SLIT3 (1 μg·mL^−1^) for 7 days. Von Kossa staining was performed to detect resorptive pit areas. Representative images of the whole well are shown in the left panel. Quantification of the resorptive area relative to the whole well area is shown in the right panel (*n* = 4). Values represent the mean ± s.e.m., n.s. (not statistically significant); *P *<* 0.05 and **P *<* 0.01 by unpaired two-tailed Student’s *t* test (**c**) or one-way ANOVA followed by Dunnett’s test (**a**, **d**). Scale bars: **c**, **d**, 200 μm

To assess the effects of exogenous SLIT3 on osteoclastogenesis, we treated wild-type BMMs undergoing osteoclast differentiation with recombinant SLIT3 at a level that showed bioactivity in multiple other cellular assays (1 μg·mL^−1^), including tube formation assays in endothelial cells.^8,11,15^ Although SLIT3 treatment modestly inhibited the expression of osteoclast marker genes, we were not able to observe a significant change in the number of TRAP-positive osteoclasts compared with controls (Fig. 2a–c). Similar negative results were obtained during a dose titration of SLIT3 during osteoclast differentiation (Fig. 2d). Moreover, recombinant SLIT3 treatment did not affect the osteoclast activity during an in vitro mineral resorption assay (Fig. 2e). These findings are consistent with our prior report that osteoclast numbers and serum levels of crosslinked C-telopeptide of type I collagen (CTX), a marker of osteoclast activity, were not substantially altered in *Slit3*^−/−^ mice.^8^

### Deletion of *Slit3* did not affect osteoclast progenitor cells in vivo

**Fig. 3:**
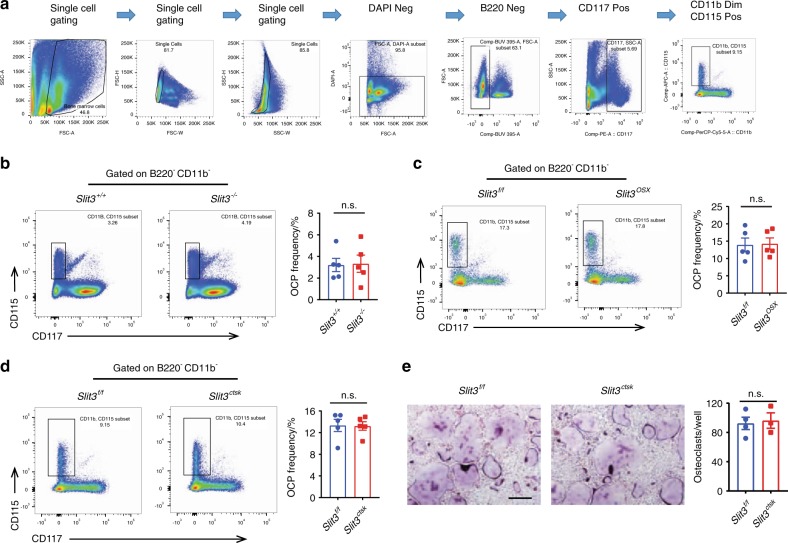
SLIT3 is dispensable for the production of osteoclast progenitors. **a** Schematic representation of the strategy used for flow cytometric analysis of osteoclast precursors. **b**–**d** Flow cytometric analysis of osteoclast precursor populations in 5-week-old male *Slit3*^*−/−*^ (**b**), *Slit3*^*osx*^ (**c**) and *Slit3*^*ctsk*^ (**d**) mice with the corresponding controls. Representative flow cytometry plots for each experiment are shown (left panels). The frequencies of osteoclast precursor populations in B220^−^- and CD11b^−^-gated BM cells from bilateral femurs and tibiae are presented (right panels) (*n* = 5). **e** Osteoclast differentiation using sorted osteoclast precursors from 5-week-old male *Slit3*^*ctsk*^ mice (*n* = 4) and littermate controls (*n* = 3) stimulated with RANKL for 72 h. TRAP staining (left panel) was performed, and the area of TRAP-positive cells (≥3 nuclei/cell) per well relative to the WT control was calculated (right panel). Values represent the mean ± s.e.m., n.s. (not statistically significant) by unpaired two-tailed Student’s *t* test. Scale bar: 200 μm

To assess the role of SLIT3 in osteoclastogenesis in vivo, we first analyzed CD117^+^CD11b^dim^CD115^+^ osteoclast precursors (OCPs) in male *Slit3*^*−/−*^ mice using flow cytometry and found no apparent differences in frequency (Fig. 3a, b),^20^ despite the severe osteopenic phenotype observed in these mice. Next, as osteoblast-derived SLIT3 is crucial for bone mass accrual, we asked whether osteoblast-derived SLIT3 would specifically affect osteoclast precursors. To this end, we bred *Slit3*^*f/f*^ mice to the Osx-cre deleter strain in which osteoblasts were targeted (*Slit3*^*osx*^). Similar to observations made following the global deletion of SLIT3, the number of osteoclast precursors in male *Slit3*^*osx*^ mice was unaltered despite the severe osteopenia observed in these mice^8^ (Fig. 3c). Finally, to confirm the role of SLIT3 in osteoclasts, we bred *Slit3*^*f/f*^ mice to a cre deleter strain in which mature osteoclasts were targeted, CTSK-cre (*Slit3*^*ctsk*^) mice. No apparent difference in the overall abundance of osteoclast precursors in male *Slit3*^*ctsk*^ mice was observed, indicating that the deletion of SLIT3 in CTSK-positive cells did not disrupt osteoclast precursors (Fig. 3d). Furthermore, fluorescence-activated cell sorting (FACS)-isolated osteoclast precursors derived from *Slit3*^*ctsk*^ mice displayed intact osteoclast differentiation capacity in vitro (Fig. 3e). Taken together, these results indicate that SLIT3 is dispensable for the generation and homeostasis of osteoclast progenitors.

### Bone mass and skeletal vasculature are normal in *Slit3*^*ctsk*^ mice

**Fig. 4:**
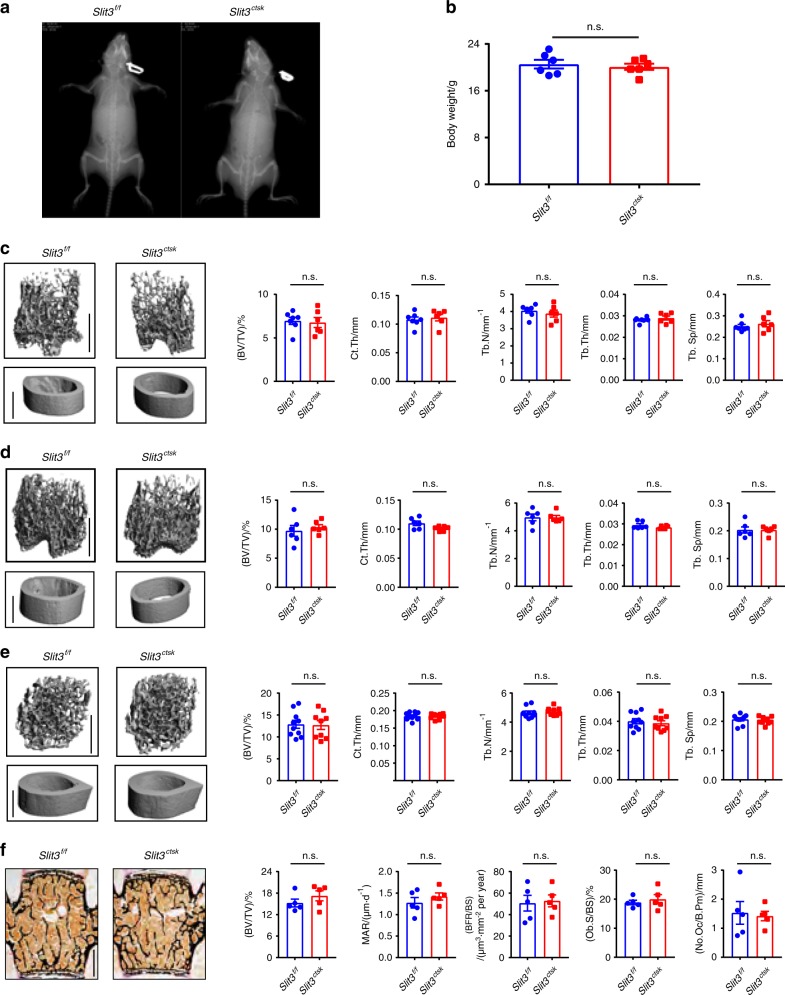
Conditional deletion of SLIT3 in CTSK-positive cells results in normal bone volume and normal osteoclast numbers in vivo. **a**, **b** Representative radiographic images (**a**) and body weights (**b**) of 5-week-old male *Slit3*^*ctsk*^ mice and *Slit3*^*f/f*^ mice (n = 6). **c**–**e** Representative micro-CT images, femoral BV/TV, cortical bone thickness, and bone morphometric analysis of trabecular bone of the distal femurs isolated from littermate *Slit3*^*ctsk*^ mice and *Slit3*^*f/f*^ mice. **c** 5-week-old female *Slit3*^*ctsk*^ mice (*n* = 6) and littermate controls (*n* = 7); **d** 5-week-old male *Slit3*^*ctsk*^ mice (*n* = 6) and littermate controls (*n* = 6); **e** 4-month-old male *Slit3*^*ctsk*^ mice (*n* = 9) and littermate controls (*n* = 10). **f** Representative images of histological (left panel) and histomorphometric analysis (right panel) of the L3 vertebrae in male *Slit3*^*ctsk*^ and *Slit3*^*f/f*^ mice at 4 months of age (*n* = 5). Values represent the mean ± s.e.m., n.s. (not statistically significant) by an unpaired two-tailed Student’s *t* test. Scale bars: **c**–**e**, 1 mm; **f**, 500 μm

**Fig. 5:**
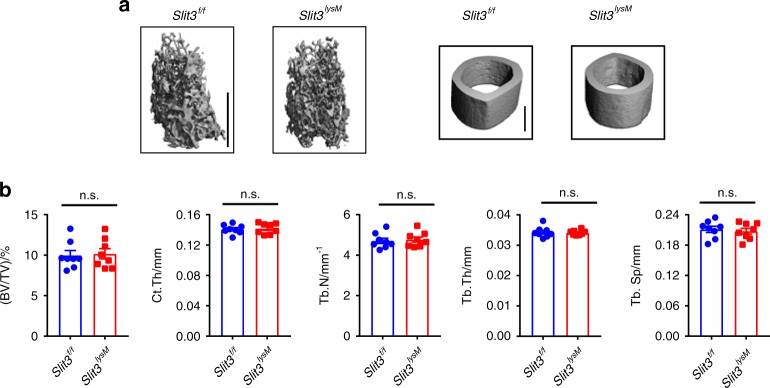
Conditional deletion of SLIT3 in LysM-positive cells results in normal bone volume. **a**, **b** Representative micro-CT images (**a**), femoral BV/TV, cortical bone thickness, and bone morphometric analysis of trabecular bone of the distal femurs (**b**) from 2-month-old female *Slit3*^*lysm*^ mice and *Slit3*^*f/f*^ mice (*n* = 8). Values represent the mean ± s.e.m., n.s. (not statistically significant) by an unpaired two-tailed Student’s *t* test. Scale bars, 1 mm

In contrast with the growth retardation and perinatal lethality observed in *Slit3*^*−/−*^ and *Slit3*^*osx*^ mice, *Slit3*^*ctsk*^ mice developed normally without any gross abnormality or decrease in body weight (Fig. 4a, b). Micro-CT showed that neither male nor female *Slit3*^*ctsk*^ mice displayed detectable alterations in bone mass in either the trabecular or cortical compartments of long bones at 5 weeks of age relative to their littermate controls (Fig. 4c, d). We further examined 4-month-old male *Slit3*^*ctsk*^ mice versus littermate controls and also observed no detectable skeletal phenotype (Fig. 4e). Consistent with this finding, histomorphometric analysis of the vertebral bone showed that the mineralization rate, bone formation rate, and numbers of osteoblasts and osteoclasts were also not affected in *Slit3*^*ctsk*^ mice. (Fig. 4f). In addition, in contrast to the vascular phenotype observed in *Slit3*^−/−^ and *Slit3*^osx^ mice, neither the visualization of endothelial cells with CD31 or EMCN immunofluorescence nor endothelial cell flow cytometry detected vascular alterations in *Slit3*^*ctsk*^ mice (Supplementary Fig. 1a–c).^8,9^ Taken together, these data indicate that the deletion of SLIT3 in CTSK-positive osteoclasts was unable to alter bone remodeling and skeletal vascular endothelium, in contrast to the strong effects on both of these parameters in osteoblast lineage cells observed with the deletion of SLIT3. Finally, to further evaluate the role of SLIT3 in osteoclast lineage cells, we bred *Slit3*^*f/f*^ mice to the cre deleter strain LysM-cre in which osteoclasts were targeted in addition to macrophages and neutrophils (*Slit3*^*lysM*^).^21^ Similar to observations in *Slit3*^*ctsk*^ mice, female *Slit3*^*lysM*^ mice at 8 weeks of age did not show a discernible change in bone mass accrual (Fig. 5a, b).

### Ablation of SLIT3 in early osteoclast lineage cells leads to normal bone mass accrual

**Fig. 6:**
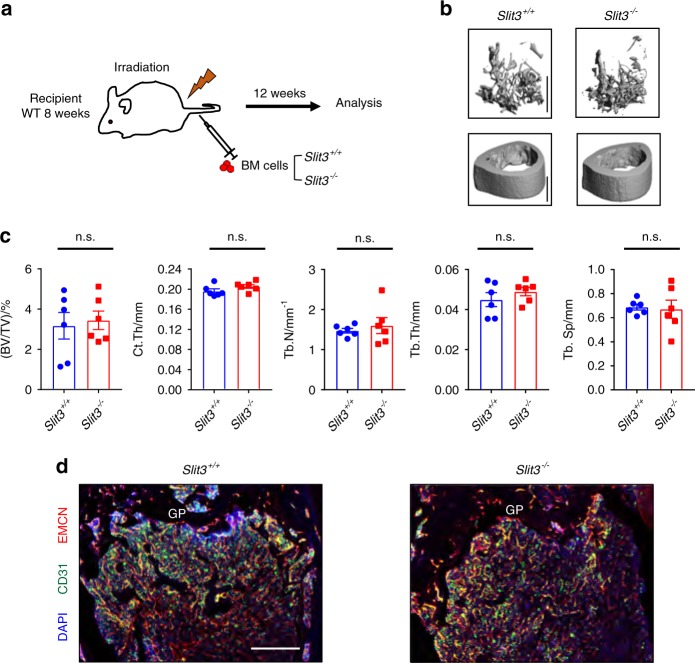
Ablation of SLIT3 in hematopoietic cells exhibits a normal bone phenotype and skeletal vasculature. **a** Schema showing the protocol for BM transplantation. Representative micro-CT images (**b**), femoral BV/TV, cortical bone thickness, and bone morphology of the distal femur trabecular bone (**c**) and representative confocal images of DAPI, CD31, and EMCN fluorescence in femur sections (**d**) isolated from bone marrow chimeras created using 5-week-old *Slit3*^*+/+*^ and *Slit3*^*−/−*^ mouse donors and WT hosts (*n* = 6). GP growth plate. Values represent the mean ± s.e.m., n.s. (not statistically significant) by unpaired two-tailed Student’s *t* test. Scale bars, 500 μm

As CTSK-cre mediates gene deletion relatively late in osteoclast differentiation,^22^ we created bone marrow chimeras from *Slit3*^*−/−*^ donors to assess the importance of SLIT3 production by early osteoclast lineage cells.^23^ Bone marrow was collected from 6-week-old male *Slit3*^*+/+*^ mice and *Slit3*^*−/−*^ mice and then injected into lethally irradiated 6-week-old male WT mice. A CD45.1/CD45.2 congenic system was used to validate the efficiency of hematopoietic reconstitution 8 weeks after bone marrow transplantation. Flow cytometric analysis showed that B220^+^ cells, Gr1/CD11b^+^ myeloid cells and osteoclast precursors all showed nearly 100% CD45.1^+^ donor origin. CD3^+^ cells were approximately 80% CD45.1^+^ at this time point (Supplementary Fig. 2 a–c). Thus, high levels of donor chimerism were observed, particularly within the osteoclast precursor compartment most relevant to this study. The resulting chimeras were maintained for 12 weeks before the analysis outlined in Fig. 6a. Micro-CT analysis showed that the mice reconstituted with bone marrow cells isolated from *Slit3*^*−/−*^ mice had normal bone mass, unlike those reconstituted with WT bone marrow cells, as illustrated by analysis of the trabecular bone volume/total volume and cortical bone thickness (Fig. 6a–c). Similarly, no alterations could be detected in the density of the bone vasculature in the recipients of *Slit3*^−/−^ bone marrow (Fig. 6d). Consistent with this, the shRNA-mediated suppression of SLIT3 expression in BMMs had no effect on osteoclastogenesis in vitro (Supplementary Fig. 3a–c). Collectively, the results of both the conditional deletion of *Slit3* in osteoclast lineage cells and development of bone marrow chimeras lacking SLIT3 in their hematopoietic cells, including all of the osteoclast lineage cells, indicate that osteoclasts are not a physiologically relevant source of SLIT3.

## Discussion

Here, we address the discrepancies between our recent finding that osteoblasts are the major source of SLIT3 in the skeleton and another recent report nominating osteoclasts as the major source of SLIT3. In summary, multiple approaches were unable to provide evidence supporting osteoclast lineage cells as a source of SLIT3. Two separate cre lines mediating deletion in osteoclast lineage cells and bone marrow chimeras lacking SLIT3 expression in all osteoclast lineage cells displayed no detectable alteration in bone mass. These findings are in contrast to the robust phenotypes observed with either the osx-cre- or dmp1-cre-mediated deletion of SLIT3 in osteoblast lineage cells.^8^ Consistent with the absence of an in vivo function of SLIT3 in osteoclasts, we were also unable to observe evidence of SLIT3 expression in osteoclasts, a finding corroborated by other studies. Taken together, our results indicate that osteoclasts are not a physiologically relevant source of SLIT3 under the conditions examined, leaving osteoblasts as the major source of SLIT3 in bone.

We previously reported that SLIT3 mediates its osteoanabolic effects by promoting skeletal angiogenesis. As osteoclast lineage cells have independent mechanisms to regulate the skeletal vasculature through the secretion of PDGF-BB, the finding that the angiogenic functions of SLIT3 are restricted to osteoblasts is significant as it suggests that osteoblasts and osteoclasts each have orthogonal mechanisms to participate in the regulation of angiogenesis. This further opens the possibility of fundamental differences in osteoblast-regulated versus osteoclast-regulated vascular responses through the differential actions of SLIT3 versus PDGF-BB.^23^ Moreover, if SLIT3 is primarily derived from osteoblasts, it is an attractive candidate as a key mediator of the angiogenic responses induced by osteoblast-targeting anabolic agents.

In a study by Kim et al, the deletion of SLIT3 using CTSK-cre resulted in modest osteopenia at 16 weeks of age. Here, however, we did not observe a similar bone phenotype in mice of the same age and sex as those in the Kim et al. report and also did not observe phenotypes with the other approaches used here to delete SLIT3 in osteoclasts. Notably, we previously observed robust phenotypes with the cre lines used in this study.^24,25^ Some of these discrepancies may be due to differences in the *Slit*3 floxed allele mice, as the two reports utilized different mouse lines, though the deletion strategies used in each report were similar. However, the finding that bone marrow chimeras made from *Slit3*^−/−^ donors failed to display skeletal phenotypes suggests that these discrepancies are not attributable solely to any idiosyncrasies of the *Slit*3 floxed allele utilized in the present report.

We are unable to fully exclude the possibility that osteoclasts are an additional target of SLIT3 activity based on the observation of modest effects on osteoclast gene expression after SLIT3 treatment. These effects may be mediated by the low-level expression of ROBO3 in osteoclasts.^8^ However, we were unable to detect an overall effect on the endpoint of osteoclast differentiation based on the formation of TRAP^+^ multinucleated osteoclasts in vitro or the number of either osteoclast progenitors or mature TRAP^+^ osteoclasts in vivo. Similarly, we were previously unable to detect alterations in markers of osteoclast resorptive activity in *Slit3*^−/−^ or *Slit3*^osx^ mice displaying severe osteopenia. In the context of the robust angiogenic responses evoked by SLIT3 in vivo and in vitro, this finding reinforces our model that osteoblast-derived SLIT3 acts on endothelial cells to evoke osteoanabolic responses. The differences regarding the in vitro effects of SLIT3 between the report by Kim et al. and the present report may reflect differences in the utilized source of SLIT3 or other factors. Notably, in addition to the failure of exogenous SLIT3 to exert a robust effect, in vitro genetic approaches had no effect. By serving as an endothelium-targeted osteoanabolic agent, SLIT3 may have utility when combined with traditional osteoblast- or osteoclast-targeted agents. Given that increasing evidence indicates that the optimal therapy for disorders of low bone mass may require sequential or combination therapy with multiple agents, the development of osteoanabolic agents with orthogonal action at the cellular level would be highly desired.^26,27^

## Materials and methods

### Animals

OSX-Cre, LysM-Cre, CTSK-Cre, *Slit3*^*−/−*^, and *Slit3*^*f/f*^ mice were obtained or generated as described in previous studies.^8,21,25^ All mice were maintained under a standard 12 h dark–light cycle with chow ad libitum. All animal experiments were conducted according to the guidelines approved by the Weill Cornell Medical College subcommittee on animal care.

### Osteoblast and osteoclast culture

Murine osteoclast differentiation assay was set up as previously described.^28^ Briefly, the whole bone marrow cells were flushed from the femora and tibiae dissected from the mutant mice and their littermates and plated on 10 cm dish for 3 days culture in α-MEM supplemented with 10% FBS in the presence of CMG14–12 supernatant as a source of M-CSF. Subsequently, we scraped the attached BMMs and seeded at a density of 4.5 × 10^4^/cm^2^ for osteoclast formation with the stimulation of RANKL (40 ng·mL^−1^). We evaluate osteoclastogenesis using TRAP staining. Murine primary osteoblast differentiation assay was prepared as previously described.^29^

### Osteoclast activity assay

Mineral resorption pit assay was used to evaluate the osteoclast activity as previously described.^28^ Briefly, BMMs were initially seeded on 96-well Corning Osteo Assay Surface Plates (Corning, Tewksbury, MA, USA) at a seeding density of 2 × 10^4^ per well. Subsequently, osteoclast differentiation was driven by CMG14–12 supernatant and 40 ng·mL^−1^ RANKL. Once we observed mature osteoclasts, the cells on the plate were removed by adding 10% sodium hypochlorite solution. After twice PBS washing, von Kossa staining was performed to visualize resorptive pits. Light microscopic images were captured for each well and pit areas were quantified by Image J (National Institutes of Health, Bethesda, MD, USA).

### Reverse transcription and real-time PCR

Total RNA (DNA-Free) was extracted from culture cells and tissues using an RNeasy Mini Kit (Qiagen, Germantown, MD, USA) and reverse transcription was carried on by First Strand cDNA Synthesis Kit (Thermo Fisher Scientific, Waltham, MA, USA). We performed real-time PCR using Fast SYBR Green Master Mix in the QuantStudio5 Real-Time PCR System (Applied Biosystems, Foster City, CA) according to the manufacturer’s instructions. Glyceraldehyde-3-phosphate dehydrogenase (*Gapdh*) or hypoxanthine guanine phosphoribosyl transferase (*Hprt*) was used as a control for normalization. The following primers were used: *Slit3*-forward, 5ʹ-TCCAGTGTTCCTGAAGGCTCCT-3ʹ, and *Slit3*-reverse, 5ʹ-TGGCAATGCCAGGCTCCTTGTA-3ʹ; *Ctsk*-forward, 5ʹ-AAGATATTGGTGGCTTTGG-3ʹ, and *Ctsk*-reverse, 5ʹ-ATCGCTGCGTCCCTCT-3ʹ; *Dcstamp*-forward, 5ʹ-TTTGCCGCTGTGGACTATCTGC-3ʹ, and *Dcstamp*-reverse, 5ʹ-AGACGTGGTTTAGGAATGCAGCTC-3ʹ; *Nfatc1*-forward, 5ʹ-CCCGTCACATTCTGGTCCAT-3ʹ, and *Nfatc1*-reverse, 5ʹ-CAAGTAACCGTGTAGCTCCACAA-3ʹ; *Itgb3*-forward, 5ʹ-CCGGGGGACTTAATGAGACCACTT-3ʹ, and *Itgb3*-reverse, 5ʹ-ACGCCCCAAATCCCACCCATACA-3ʹ; *Calcr-*forward, 5ʹ-CTGAAGCTTGAGCGCCTGAGTC-3ʹ, and *Calcr*-reverse, 5ʹ-TGGGGTTGGGTGATTTAGAAGAAG-3ʹ; *Trap*-forward, 5ʹ-ACCAGCAAGGATTGCGAGGCAT-3ʹ, and *Trap*-reverse, 5ʹ-GGATGACAGACGGTATCAGTGG-3ʹ; *Tnfrsf11b*-forward, 5ʹ-CGGAAACAGAGAAGCCACGCAA-3ʹ, and *Tnfrsf11b*-reverse, 5ʹ-CTGTCCACCAAAACACTCAGCC-3ʹ; *Gapdh*-forward, 5ʹ-ATCAAGAAGGTGGTGAAGCA-3ʹ, and *Gapdh-*reverse, 5ʹ-GTCGCTGTTGAAGTCAGAGGA-3ʹ; and *Hprt*-forward, 5ʹ-CTGGTGAAAAGGACCTCTCGAAG-3ʹ, and *Hprt*-reverse, 5ʹ-CAAGATATCGTTGAAACGTGGA-3ʹ.

### Transcriptional expression profiling by RNA-seq analysis

We purified total RNA using an RNeasy Mini Kit (Qiagen, Germantown, MD, USA) and used true-seq RNA Library preparation kits (Illumina, San Diego, CA, USA) to purify poly-A+transcripts and generate libraries with multiplexed barcode adapters according to the manufacturer’s protocols. After the quality of all samples passed control analysis using a Bioanalyzer 2100 (Agilent, Lexington, MA, USA), we constructed RNA-seq libraries per the Illumina TrueSeq RNA sample preparation kit and carried out high-throughput sequencing using the Illumina HiSeq 4000 in the Genomics Resources Core Facility of Weill Cornell Medical College. For analysis, we used STAR (version 2.3.0e)^30^ with the default parameters to align the reads to mm9 mouse transcripts. Then we sorted and indexed the resulting bam files using SAMtools. To obtain gene counts, we applied feature counts (version 1.4.3)^31^ to sorted bam files and excluded the genes without any expression counts. We normalize gene count data using the DESeq2 (version 1.4.5) R package.^32^

### Immunoblotting

The extracts from cultured cells and total tissue were prepared using RIPA buffer or a lysis buffer [150 mmol·L^−1^ Tris-HCl (pH 6.8), 6% SDS, 30% glycerol, and 0.03% bromophenol blue] with 10% 2-ME. The lysates were subjected to 7.5% SDS-PAGE and transferred to Immobilon-P membranes (Millipore, Billerica, MA, USA). The transferred membranes were blocked with 5% skimmed milk and incubated with specific antibodies. The following primary antibodies were used: SLIT3 (1:1 000; R&D), Nfatc1 (1:1 000; BD Pharmagen), c-Fos (1:1 000; Santa Cruz), p38 (1:3 000; Santa Cruz), and HSP90 (1:2 000; Sigma). We detected protein bands using Western Lightning plus-ECL (PerkinElmer, Waltham, MA, USA).

### Skeletal analysis

The whole-body radiographs of all mice were taken by the Faxitron X-ray system. Three dimensional skeletal images and assessments of trabecular and cortical bone from distal femur were obtained by a Scanco Medical μCT 35 system, which was calibrated weekly by scanning manufacturer-provided, resin-embedded phantoms. All sample scans were performed in 70% ethanol with an isotropic voxel size of 7 μm in the condition of an X-ray tube: 55 kVp, 0.145 mA, 600 ms integration time. The threshold of trabecular bone was 211 per mille, which corresponds to ~270 mg hydroxyapatite/ccm. The threshold of cortical bone was 350 per mille, which corresponds to ~570 mg hydroxyapatite/ccm. We reduced noise in the thresholded images using a Gaussian noise filter applied for murine bone analysis.

### Histology, histomorphometry, and immunohistochemistry

The mutant mice and relative controls were subcutaneously injected with a dose of 20 mg·kg^−1^ on day 5 and 1 before scarification. Plastic embedding, sectioning, TRAP staining, toluidine blue staining and von Kossa staining were conducted as previously described.^4^ Histomorphometric analyses were carried out using the Osteomeasure Analysis System (OsteoMetrics, Atlanta, USA) as previously described^29^ Frozen sectioning and immunofluorescence staining were conducted according to a published protocol.^33^

### Cell sorting and flow cytometry analysis

For OCP frequency analysis, bone marrow cells collected from *Slit3*^*+/+*^ and *Slit3*^*−/−*^mice, *Slit3*^*f*/*f*^ and *Slit3*^*osx*^ mice, *Slit3*^*f*/*f*^ and *Slit3*^*ctsk*^ mice were flushed out and removed debris using a 70 µm cell strainer. For skeletal endothelial cell analysis, the femurs and tibias from *Slit3*^*f*/*f*^ and *Slit3*^*ctsk*^ mice were isolated, and the surrounding muscles and connective tissues were cleaned. We crushed the bones in Hank’s balanced salt solution with 10 mmol·L^−1^ HEPES. For the enzymatically digestion, 2.5 mg·mL^−1^ Collagenase A and 1 U·mL^−1^ Dispase II were added and mixed thoroughly for 15 min incubation at 37 °C with gentle agitation. Next, we added PBS containing 0.5% FBS and 2 mmol·L^−1^ EDTA to stop digestion and the resulting suspensions filtered through a 40 µm cell strainer and washed twice with PBS. After washing, antimouse CD16/CD32 antibody (BD) was used to block unspecific staining for 15 min on ice. For OCP analysis, the cells were stained with BUV395-conjugated B220 antibody (BD), PerCP-Cy5.5-conjugated CD11b (BioLegend), FITC-conjugated CD115 (BioLegend) and PE/Cy7-conjugated CD117 (BioLegend) for 30 min on ice. For skeletal endothelial cell analysis, the cells were stained with FITC-conjugated CD45 (BioLegend), APC/Cy7-conjugated Ter119 (BioLegend), PE-conjugated CD31 (eBioscience) and APC-conjugated EMCN antibody (eBioscience) for 30 min on ice. DAPI solution (1 μg·mL^−1^) was used for live/dead exclusion. Cell sorting was performed with FACS Aria II (BD, San Jose, CA, USA) and analyzed using FlowJo software (Tree Star, Ashland, OR, USA).

### Bone marrow transplantation

We followed a published protocol for bone marrow transplantation.^34^ Briefly, recipient mice (6-week-old BALB/c mice) were irradiated with a lethal dose of 875 rads 1 day before transplantation. The whole bone marrow cells from *Slit3*^*+/+*^ and *Slit3*^*−/−*^mice were harvested, and 5 million bone marrow cells per donor mouse were transplanted into each irradiated recipient via intravenous tail vein injection. The resulting bone marrow chimeras were euthanized 16 weeks after transplantation. Congenic CD45.1/CD45.2 mice were used to validate the reconstitution efficiency of bone marrow transplantation in our protocol described in Supplementary 2.

### Statistical methods

All data statistical analysis in our study was performed using GraphPad Prism software (v6.0a; GraphPad, La Jolla, CA, USA). Two-tailed Student’s *t* test was used to determine significance for only two groups compare. One-way ANOVA with Tukey’s post-hoc tests was used to determine significance for compare between multiple groups. A *P* value < 0.05 indicated statistical significance. Error bars are presented as mean ± s.e.m.

## Supplementary information

Supple 1

Supple 2

Supple 3
