# Supplementary figures and images for: Osteoclasts are not a source of SLIT3
Source: Bone Res. 2020 Feb 19;8:11. doi: 10.1038/s41413-020-0086-3 (PMC7031526; doi:10.1038/s41413-020-0086-3)

# Supplementary Fig. 1

a

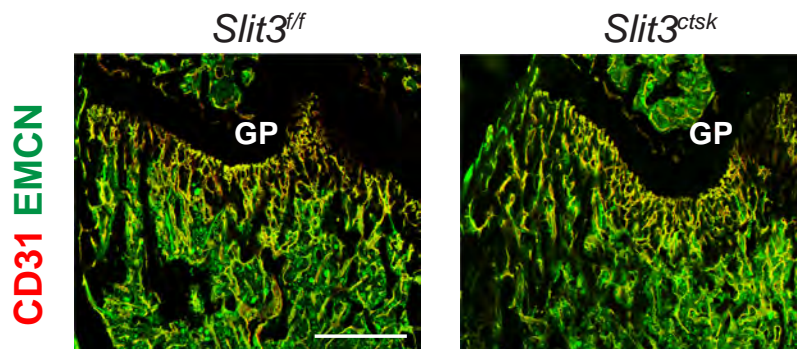

b

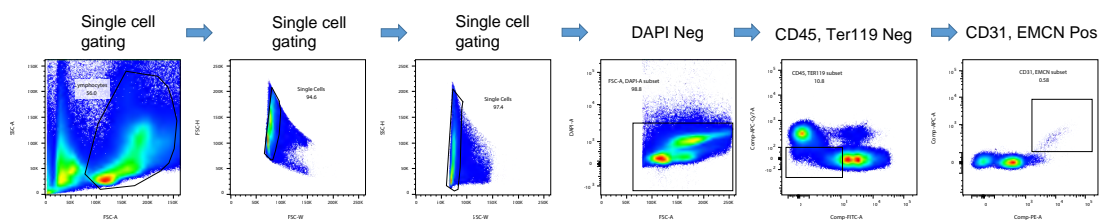

c

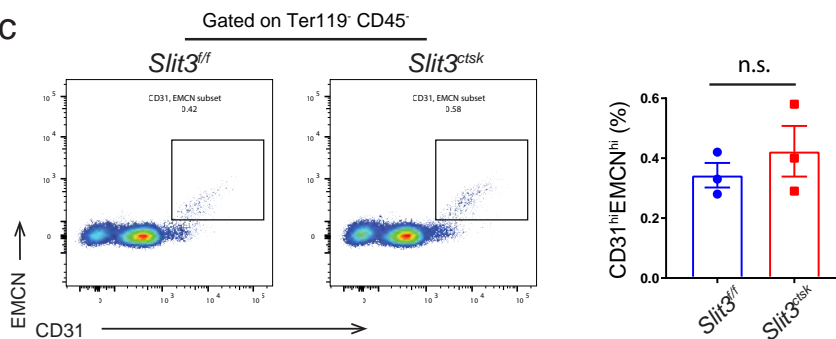

Supplement: Supplementary file 1 — Supple 1 [file 41413_2020_86_MOESM1_ESM.pdf]

# Supplementary Fig. 2

a

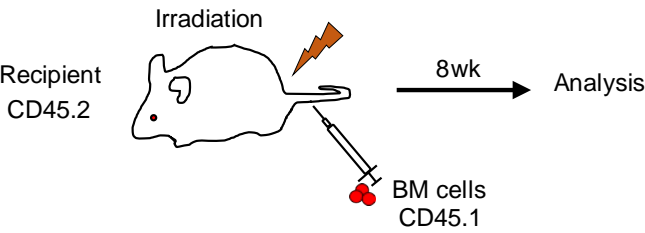

b

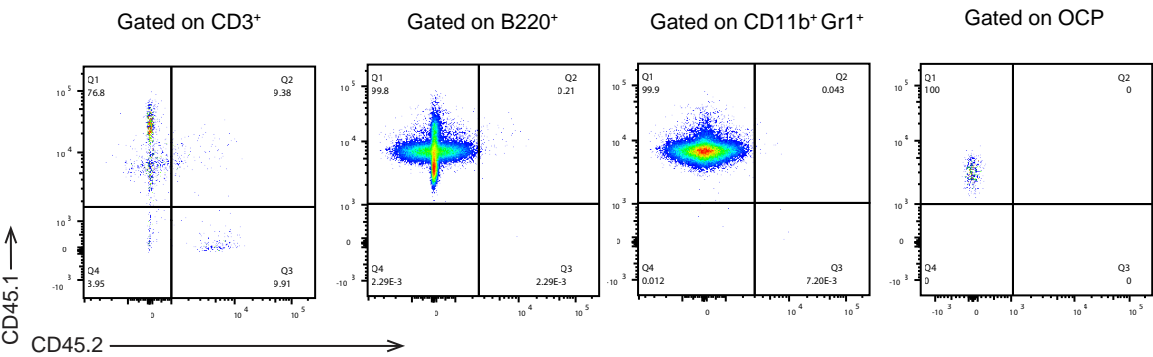

c

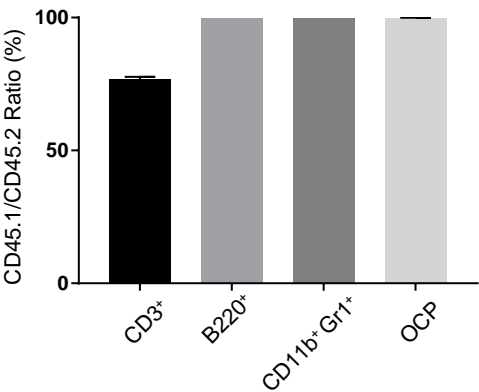

Supplement: Supplementary file 2 — Supple 2 [file 41413_2020_86_MOESM2_ESM.pdf]

# Supplementary Fig. 3

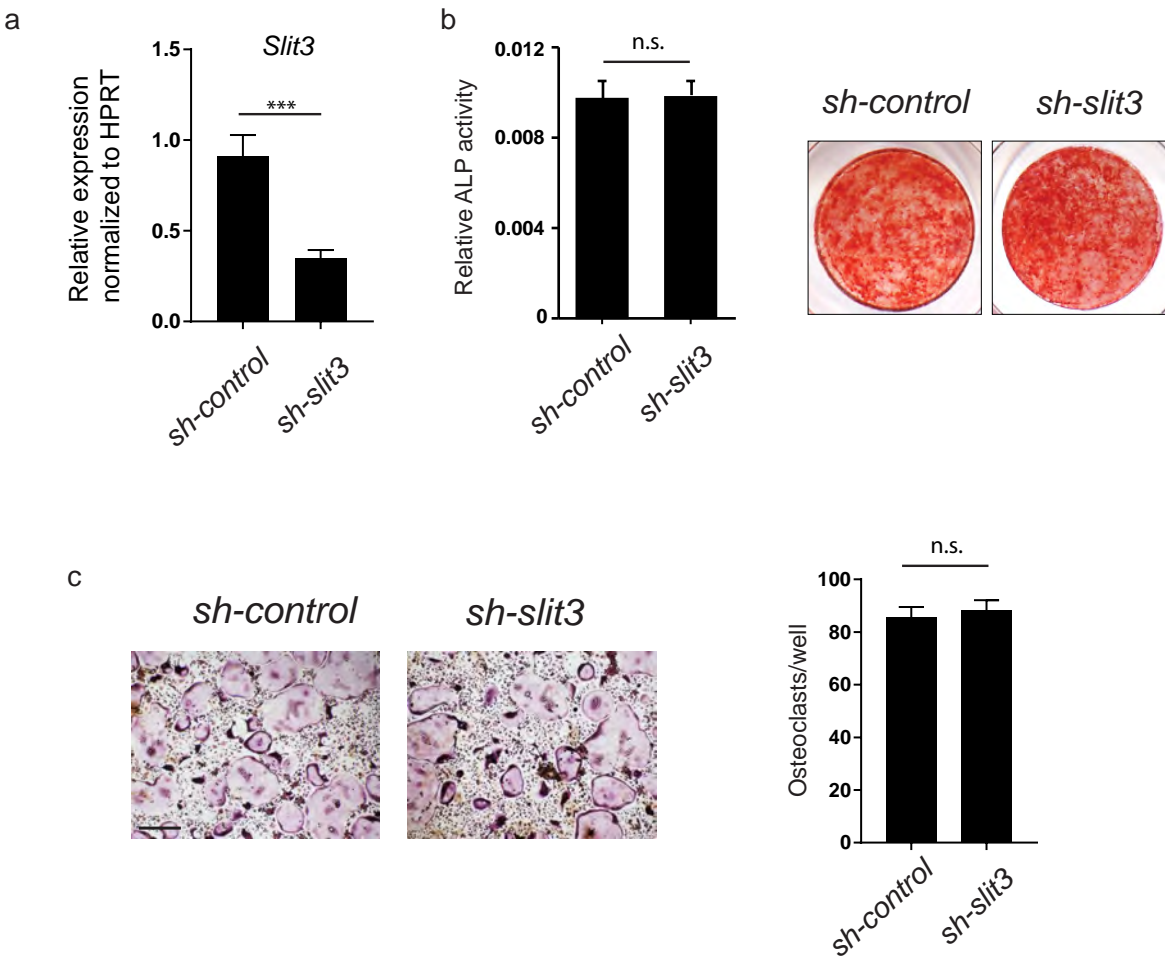

Supplement: Supplementary file 3 — Supple 3 [file 41413_2020_86_MOESM3_ESM.pdf]
